# T4 Erector Spinae Plane Block Relieves Postdural Puncture Headache: A Case Report

**DOI:** 10.7759/cureus.6237

**Published:** 2019-11-26

**Authors:** Johanna B De Haan, Olga M Chrisman, Linden Lee, Michelle Ge, Nadia Hernandez

**Affiliations:** 1 Anesthesiology, McGovern Medical School University of Texas Health Science Center, Houston, USA

**Keywords:** erector spinae plane block, postdural puncture headache, ultrasound guided block, peripheral nerve block, headache nerve block, regional block, obstetric analgesia, post-dural puncture headache, spinal headache

## Abstract

Postdural puncture headache (PDPH) is a common complication of neuraxial anesthesia. The gold standard treatment for PDPH is an epidural blood patch (EBP). However, the risks of EBP, and patient willingness to undergo another attempted neuraxial procedure, can prevent patients from receiving this treatment. The erector spinae plane (ESP) block has been used in the treatment of acute postoperative and chronic pain secondary to many indications at many vertebral levels, and a prior case series describes two patients in which ESP block relieved tension headache. In our case report, we describe a novel use of the ESP block at the fourth thoracic vertebral level to relieve PDPH in a super morbidly obese patient with two prior inadvertent dural punctures.

## Introduction

Postdural puncture headache (PDPH) is a common complication associated with neuraxial procedures, particularly in the obstetric population. Dural punctures during epidural placement occur with a frequency of about 1.5%, and 50% of those patients develop a clinically significant PDPH [1]. PDPH can be debilitating and result in uncontrolled pain with upright positions, increased opioid use and incidence of postpartum depression, reduced postpartum mobility, maternal-neonatal bonding and ability to breastfeed [2]. Multiple treatment modalities exist for symptomatic relief of PDPH, but the epidural blood patch (EBP) is considered the gold standard of treatment with an efficacy of up to 98% [3]. However, the EBP is an invasive procedure that has inherent risks. Notably, patients who had an unintentional dural puncture during their first neuraxial procedure are at risk for a second inadvertent dural puncture when undergoing EBP [3]. Other complications of EBP have been reported, including arachnoiditis, spinal and epidural hematoma, seizures, cerebral venous sinus thrombosis, cranial nerve palsies, and intracerebral hemorrhage [2-3]. Due to these risks, patients may opt for conservative management or alternative treatments [4]. Other treatment modalities for PDPH have been explored, including peripheral nerve blocks. Transnasal sphenopalatine ganglion (SPG) block demonstrated relief of symptoms of PDPH in 71.4% of patients; however, the literature on its efficacy is limited to one retrospective review and a few case reports [5]. In addition, transnasal placement of local anesthetic applicators may make this option undesirable to some patients. The greater occipital nerve block (GONB) has been used, with 66% efficacy, and occasionally requiring multiple injections to achieve and sustain analgesia [6-7]. The erector spinae plane (ESP) block was recently described in 2016 by Forero et al., and many uses of the block have been reported to treat both acute and chronic pain [8]. Ueshima et al. reported two cases in which a high thoracic ESP block relieved tension headache [9]. We describe a case where a high thoracic ESP block relieved the symptoms of PDPH in a super morbidly obese patient with two prior inadvertent large-gauge dural punctures.

## Case presentation

A super morbidly obese (body mass index 57) 34-year-old primiparous parturient presented at 39 weeks and six days gestation for induction of labor. Challenging lumbar epidural placement resulted in two inadvertent dural punctures with a 17-gauge Tuohy needle. The patient subsequently had successful epidural placement, and underwent Cesarean delivery due to the arrest of cervical dilation. On postpartum day (PPD) two, she reported a fronto-occipital headache that she rated as a ten out of ten on a numeric pain scale (NPS). The headache was worsened by sitting up or standing, and her symptoms were relieved by lying flat. Because she was hesitant to undergo another neuraxial procedure, the acute pain service was consulted for analgesic alternatives to EBP. The option of an ultrasound-guided T4 ESP block was offered to the patient, with the caveat that its efficacy for relief of PDPH was unknown, as it had only been reported to relieve tension headache. 

**Figure 1:**
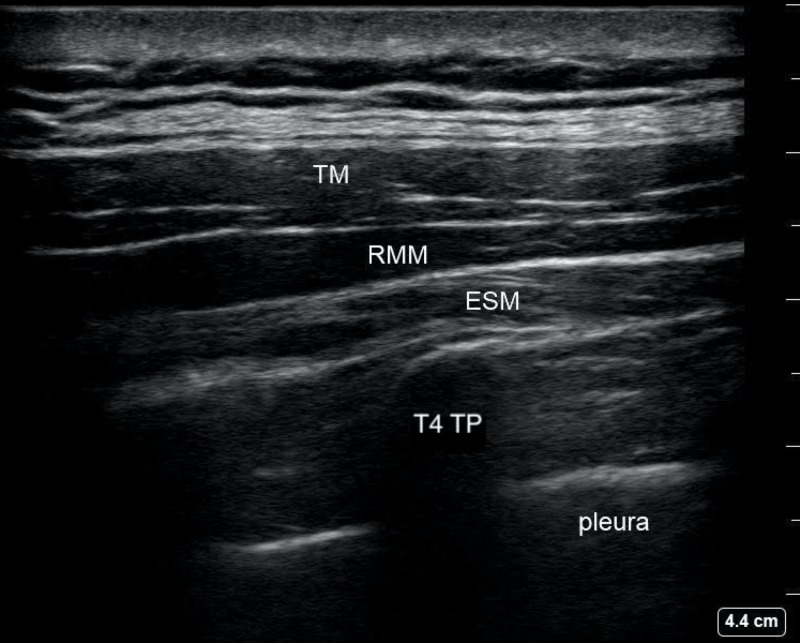
T4 Erector Spinae Plane Block Ultrasound Anatomy TM: trapezius muscle; RMM: rhomboid major muscle; ESM: erector spinae muscle; T4 TP: fourth thoracic vertebrae transverse process

The patient was transferred from the postpartum floor to the block room where standard monitors were applied, and emergency equipment and rescue medications were readily available. She was sedated with intravenous midazolam 4 mg and fentanyl 100 mcg, and placed in the left lateral decubitus position. A curvilinear ultrasound probe was placed in a parasagittal plane over the fourth rib between the spinous processes and the scapula. The fourth rib was traced medially until the costovertebral junction, transverse process (TP), erector spinae muscles (ESM), and pleura were visualized (Figure 1).

Following skin sterilization, a 22-gauge blunt-tipped, 100 mm echogenic block needle was advanced in-plane to make osseous contact with the transverse process of the fourth thoracic vertebra (TP) under direct ultrasound-guidance. A corkscrew motion of the needle was used to advance the needle tip into the fascial plane between the ESM and the TP. Then, 20 mL of 0.25% bupivacaine hydrochloride with 3 mg of preservative-free dexamethasone was injected in 5 mL increments, aspirating to ensure negative aspiration of blood or air. The procedure was repeated on the contralateral side in the same position. 

The patient was monitored for 30 minutes following the block, and at that time she reported complete resolution of the headache as she was being transported back to her room in a sitting position. Three hours later, the patient rated her pain score as a three out of ten on the Neuropathic Pain Scale (NPS) localized to her Cesarean incision site, with complete resolution of her headache. On PPD three, the patient continued to be headache-free and she was discharged home. The patient denied recurrence of headache and reported good functionality during follow-up phone call on PPD four. She consented to publication.

## Discussion

High thoracic ESP block has previously been reported to relieve tension headache in two patients [9]. This is the first report of ESP block relieving the symptoms of PDPH. In this super morbidly obese patient with symptoms indicative of PDPH who refused EBP, the options of peripheral nerve blocks such as GONB, SPG block, and ESP block were discussed. Due to the low efficacy of GONB and the possibility of requiring repeat injections to completely relieve pain, as well as reported adverse effects of GONB, such as transient cranial nerve palsies, nausea, syncope, dizziness, and potential hair loss and skin thinning in area of repeat injection, the patient declined [6-7]. The risks and benefits of the SPG block were discussed and the patient preferred to avoid intranasal instrumentation. Due to the stressful and traumatic nature of her initial neuraxial procedure for labor analgesia, the patient refused EBP. Despite reported complications of ESP block including pneumothorax and the potential for block failure, the patient opted to receive a high thoracic ESP block under sedation. 

The ESP block was first described and used for the treatment of neuropathic thoracic pain by Forero et al. in 2016 [8]. Cadaveric and CT imaging studies were used to analyze the spread of local injection of dye and contrast at the transverse process of T5 between the rhomboid major and ESM as well as deep to the ESM. This study observed the spread of injections to dorsal and ventral rami of spinal roots [7]. Dexamethasone was added to prolong the duration of the block. ESP has since been used to relieve acute post-operative pain in the neck, shoulder, thorax, abdomen, and lower extremities with a variety of vertebral level injection points [10-16]. Ueshima et al. reported two cases in which T4 ESP block relieved tension headache [9]. Identification of anatomic structures necessary to perform ESP block is easily done with ultrasound. Although bleeding can occur with any invasive procedure, there have not been any reported cases of clinically significant bleeding following ESP block [17]. Nerve injury is not an expected complication of the ESP block. There have been reports of pneumothorax with ESP block, however, if the block is performed by a physician proficient in ultrasound-guided regional anesthesia, and the needle tip is visualized at all times, this risk can be greatly minimized [18]. 

We propose the following theory to explain the analgesia obtained by a high thoracic ESP block for headache. Forero et al. demonstrated radiopaque contrast dye tracking up to the third cervical vertebra (C3) two hours following injection at the T5 level [10]. By reaching the upper cervical nerve roots with the spread of local anesthetic injectate, it is possible that the trigeminocervical complex is also affected by the local anesthetic. The trigeminocervical complex has been well studied, particularly in the context of migraine headaches. It is described as the convergence of afferent signals from the trigeminal ganglia and the upper cervical nerve roots. This convergence has been used to explain the phenomenon of referred pain from the cervical region to the frontal cranial region and vice versa. For instance, tumors in the posterior fossa, direct cervical nerve root stimulation, direct infratentorial dural stimulation, vertebral artery dissection, and noxious stimuli of tissue innervated by the greater occipital nerve, which originates mainly from the second cervical spinal nerve (C2), can all manifest as frontal head pain [19]. In animals, the trigeminocervical complex has been demonstrated to extend from the trigeminal nucleus caudalis to C2 and C3 [20]. It has been established that nociceptive signals from the dura mater and meninges are transmitted via the V1 branch of the trigeminal nerve and synapse with second order neurons in the trigeminocervical complex [20]. It would, therefore, be plausible that pain caused by meningeal traction in PDPH could be relieved by blocking the upper cervical nerve roots via the trigeminocervical complex. This implies other promising potential analgesic targets for the high thoracic ESP block besides those that have been recently reported in the literature.

## Conclusions

While EBP remains the gold standard treatment for PDPH, ESP blocks may be an alternative treatment to achieve relief of PDPH that can be performed while lying flat, under sedation, and does not put the patient at risk for a second dural puncture. It should be noted that ESP block for PDPH may provide symptomatic relief of headache, but will not remedy the pathology causing PDPH. Additional investigation into this topic will help to further evaluate high thoracic ESP block efficacy and reproducibility for symptomatic relief of PDPH.
